# Editorial: Empowering early career researchers in psychiatry: advancing autism research

**DOI:** 10.3389/fpsyt.2025.1718609

**Published:** 2025-11-17

**Authors:** Angela Caruso, Célia Rasga, Francesca Fulceri, Maria Luisa Scattoni, Martina Micai

**Affiliations:** 1 Coordination and Promotion of Research Service, Istituto Superiore di Sanità, Rome, Italy; 2 Instituto Nacional De Saúde Doutor Ricardo Jorge, Lisbon, Portugal

**Keywords:** autism spectrum disorder, early career, neurodevelopment, family intervention, cognition

This Research Topic, entitled *“Empowering Early Career Researchers in Psychiatry: Advancing Autism Research,”* aims to highlight the contributions of early career researchers (ECRs) who are shaping the future of autism research. Autism research is a rapidly evolving field, with continuous advances in the understanding of the genetic, environmental, and neurobiological mechanisms underlying autism spectrum disorder (ASD), along with the development of early intervention strategies and personalized approaches in this field. Nevertheless, despite these advancements, important gaps remain in knowledge, practice, and translation, making the perspectives of ECRs particularly valuable.

Globally, autism spectrum disorder (ASD) affects approximately 1 in 100 children, with notable regional variations in prevalence and diagnostic recognition. This global perspective underscores the importance of culturally sensitive research and highlights the value of contributions from diverse regions.

Early career researchers often bring fresh insights, methodological innovations, and cross-disciplinary approaches that are crucial to addressing the complexities of autism and improving outcomes for autistic individuals. This Research Topic provides a platform to showcase their innovative work and to emphasize the importance of supporting the next generation of clinicians and scientists in the field of psychiatry and autism research.

The Research Topic comprises six articles authored by early-career researchers from diverse backgrounds and institutions. Specifically, it consists of one study protocol, four original research articles, and one review article.

## 1 Epidemiology and global perspectives

The study by Nukeshtayeva et al. examined epidemiological trends of ASD and other neurodevelopmental disorders (NDDs) in Kazakhstan between 2016 and 2022. Drawing on national healthcare databases, the authors analyzed the incidence and prevalence of childhood autism, atypical autism, and broader NDDs across different regions of the country. Their findings reveal a nearly fivefold increase in childhood autism diagnoses and a fourfold increase in atypical autism over this seven-year period, both of which are statistically significant. In contrast, no consistent trend was observed for other NDDs. Regional variation was evident, with northern Kazakhstan reporting higher ASD rates than southern regions, a difference attributed to disparities in healthcare infrastructure, urbanization, and environmental factors, such as pollution. These results point to a significant public health shift in Kazakhstan, reflecting both rising incidence and improved diagnostic recognition of ASD.

## 2 Cognitive and neurobiological mechanisms

Within the domain of cognition and learning in ASD, recent studies have examined different aspects of cognitive processing in children. For example, Wang et al. focused on the neural mechanisms of spatial navigation in children with ASD using EEG microstates and theta-band functional connectivity during a map-reading task. Compared to their typically developing peers, children with ASD showed altered microstate dynamics, including a longer duration of microstate A, reduced stability in microstates B and C, and an increased occurrence of microstate D, reflecting atypical spatial attention. Connectivity analyses revealed reduced theta-band communication between fronto-parietal and occipito-temporal regions, indicating disrupted integration of spatial and cognitive processes. These findings suggest that microstate and connectivity measures may serve as potential biomarkers of spatial navigation deficits in ASD, supporting the development of individualized rehabilitation strategies. Bejarano-Martín et al. investigated early numerical skills and mathematical performance in primary school children with ASD compared to typically developing peers. Forty-two children (21 with ASD and 21 typical development, TD) were assessed on control tasks and in the domains of early numerical skills (counting, subitizing, magnitude comparison, and estimation) and mathematical domains (arithmetic calculation and word problems). The results showed significant differences in subitizing and estimation, while both groups performed similarly in arithmetic tasks. For autistic children, non-symbolic skills predicted mathematical performance, whereas symbolic skills were predictive for TD peers. These findings suggest that mathematics is not a major area of difficulty for autistic children; however, longitudinal studies are needed to further explore early numerical and mathematical development.

Together, these cognitive studies underscore the heterogeneity of learning profiles in children with ASD and highlight the need for longitudinal designs that can clarify developmental trajectories. These articles also bridge mechanistic insights with applied perspectives, pointing to avenues for individualized educational and therapeutic strategies.

## 3 Clinical assessment and diagnostic tools

Jarvers et al. examined the discriminative ability of the Short-Story Task (SST) to identify autistic individuals within outpatient autism services. The study included 211 participants, 100 of whom were diagnosed with ASD and 111 of whom were diagnosed with other conditions or none at all. Overall performance on the SST did not reliably distinguish between the groups, but linear regression analysis showed that an autism diagnosis significantly predicted SST mentalizing performance. Moreover, specific SST items differed between autistic and non-autistic participants and were predictive of diagnosis. These findings suggest that, while the SST alone may not serve as a definitive diagnostic tool, it provides useful insights into social-cognitive processing and how individuals interpret the broader context of stories.

## 4 Family-centered interventions

Dai et al. described a quasi-experimental study protocol for a nurse-led parental self-efficacy promotion program for parents of children with ASD. The study will recruit 80 parents at a tertiary hospital in China, with participants assigned to either a control group receiving routine care or an intervention group receiving a one-month program including one face-to-face session, three online sessions, and a written pamphlet. The program focuses on goal setting, experience sharing, verbal encouragement, and positive emotion mobilization. Outcomes will include parenting self-efficacy, parenting stress, intervention compliance, and family quality of life. This protocol highlights the potential of nurse-led programs to empower parents and enhance early family-based interventions for children with ASD.

Meng et al. conducted a bibliometric analysis of research on family interventions for children with ASD from 1987 to 2024. Using CiteSpace to analyze 1,891 publications, the study mapped countries, institutions, authors, journals, and research trends. The United States led in publications, followed by Canada and Australia, with Baranek as the most prolific author, and the University of California System as the top institution. Research hotspots centered on early family-based interventions and parental psychological stress. Telemedicine and parent-mediated interventions are currently the most prominent approaches, and future directions may involve applying artificial intelligence techniques to ASD interventions in home and computer-based contexts. This analysis highlights key contributors and emerging trajectories in family-centered autism research.

## 5 Conclusion

In conclusion, this Research Topic highlights the diverse contributions of early-career researchers to the field of autism research, spanning epidemiology, cognitive and neurobiological mechanisms, clinical assessment, and family-centered interventions. Collectively, these studies underscore the importance of integrating multiple perspectives—from neural and cognitive processes to family support and early intervention strategies—to advance our understanding and improve outcomes for individuals with ASD. By showcasing innovative methodologies, novel findings, and emerging research trends, this Research Topic emphasizes the critical role of early-career researchers in shaping the future of autism research and fostering evidence-based, multidisciplinary approaches in psychiatry.

Looking forward, future autism research will greatly benefit from longitudinal studies that follow individuals across the lifespan, the integration of emerging technologies such as artificial intelligence and big data analytics, and increased attention to cultural and socioeconomic diversity in study populations. Equally important will be the translation of research findings into public health policies and clinical practice to ensure that discoveries ultimately improve the lives of autistic individuals and their families worldwide.

